# Comparative Genomics and CAZyme Genome Repertoires of Marine *Zobellia amurskyensis* KMM 3526^T^ and *Zobellia laminariae* KMM 3676^T^

**DOI:** 10.3390/md17120661

**Published:** 2019-11-24

**Authors:** Nadezhda Chernysheva, Evgeniya Bystritskaya, Anna Stenkova, Ilya Golovkin, Olga Nedashkovskaya, Marina Isaeva

**Affiliations:** 1 G.B. Elyakov Pacific Institute of Bioorganic Chemistry, Far Eastern Branch, Russian Academy of Sciences, 159, Pr. 100 let Vladivostoku, Vladivostok 690022, Russia; chernysheva.nadezhda@gmail.com (N.C.); belyjane@gmail.com (E.B.); oined2012@gmail.com (O.N.); 2 Far Eastern Federal University, 8 Sukhanova St., Vladivostok 690090, Russia; stenkova@gmail.com (A.S.); golovkin.io.1996@gmail.com (I.G.)

**Keywords:** marine flavobacteria, *Zobellia*, comparative genomics, carbohydrate-active enzymes

## Abstract

We obtained two novel draft genomes of type *Zobellia* strains with estimated genome sizes of 5.14 Mb for *Z. amurskyensis* KMM 3526^Т^ and 5.16 Mb for *Z. laminariae* KMM 3676^Т^. Comparative genomic analysis has been carried out between obtained and known genomes of *Zobellia* representatives. The pan-genome of *Zobellia* genus is composed of 4853 orthologous clusters and the core genome was estimated at 2963 clusters. The genus CAZome was represented by 775 GHs classified into 62 families, 297 GTs of 16 families, 100 PLs of 13 families, 112 CEs of 13 families, 186 CBMs of 18 families and 42 AAs of six families. A closer inspection of the carbohydrate-active enzyme (CAZyme) genomic repertoires revealed members of new putative subfamilies of GH16 and GH117, which can be biotechnologically promising for production of oligosaccharides and rare monomers with different bioactivities. We analyzed AA3s, among them putative FAD-dependent glycoside oxidoreductases (FAD-GOs) being of particular interest as promising biocatalysts for glycoside deglycosylation in food and pharmaceutical industries.

## 1. Introduction

Seaweeds are a rich source of bioactive compounds particularly with regard to polysaccharides. Red seaweeds (*Rhodophyceae*) produce sulfated galactans, such as agar and carrageenan. Other sulfated polysaccharides, such as ulvans or fucans, are found in green (*Chlorophyceae*) or brown (*Phaeophyceae*) seaweeds, respectively. Non-sulfated polysaccharides, mainly laminarans and alginates, are isolated from brown seaweeds. These polysaccharides are being actively studied due to their pharmacological anti-inflammatory, antioxidant, antiviral, antitumor, immunomodulatory, anticoagulant, hypolipidemic, and prebiotic activities [1,2]. Physical-chemical properties and biological activities of their derivatives are of great interest for study. Previous works showed they have the potential to be used as bioactive molecules and functional materials in food, pharmaceutical, and cosmetic industries [3,4,5]. Among seaweed polysaccharides, agar and carrageenan are valuable sources of various oligosaccharides with beneficial effects for human health, and these effects depend on the degree of depolymerization [6]. The oligosaccharides, in turn, are a source of rare sugars, such as 3,6-anhydro-l-galactose (L-AHG), which has been recently suggested to be a new anticariogenic sugar [7]. Importantly, AHG-containing oligosaccharides have been reported to demonstrate anti-inflammatory, antitumor, and anticariogenic activities [8,9,10]. They can be also used in cosmetic dermatology for skin moisturizing and whitening [11,12].

The most eco-friendly methods for improving the yield and quality of algal polysaccharides and their derivatives are enzyme-based techniques [1,4]. Therefore, there is a demand for highly specific hydrolytic enzymes, which in turn stimulates the search for marine bacteria specialized in the degradation of various polysaccharides. Bacterial carbohydrate-active enzymes (CAZymes) are responsible for synthesis and degradation of polysaccharides as well as their derivatives [13]. They include glycoside hydrolases (GHs), glycosyltransferases (GTs), polysaccharide lyases (PLs), and carbohydrate esterases (CEs). Now, they also include auxiliary activity (AAs) enzymes and carbohydrate-binding modules (CBMs). CAZymes have been successfully used in biotechnological, medical, and industrial applications [14]. It is necessary to take into account that the CAZyme repertoire of microorganisms might be determined by both the taxonomic level and ecological niche they occupy [15]. Therefore, a comparative genomics approach provides insights into a “core” CAZome that is conserved among organisms and an organism-specific “accessory” CAZome that encodes uniquely for each particular organism enzyme.

The phylum Bacteroidetes accommodates bacteria distributed across diverse habitats, including terrestrial, aquatic, and gut ecosystems [16,17,18,19,20]. Marine representatives of the Bacteroidetes are involved in many biogeochemical processes and specialize in the degradation of various biopolymers [20] due to their metabolic flexibility and special enzymatic repertoires [21]. It is known that Flavobacteriia, the most numerous class of the phylum, are specialized in the degradation of algal polysaccharides [22,23,24,25]. To date, genome investigations of marine Flavobacteriia, such as *Gramella forsetii* KT0803^T^ [26], *Cellulophaga algicola* IC166^T^ [27], *Polaribacter* sp. Hel1_85 [28], *Formosa agariphila* M-2Alg 35-1^T^ [29], and *Zobellia galactanivorans* Dsij^T^ [30], have revealed an abundance of CAZyme genes, confirming their specialization in the utilization of polysaccharides in marine environments.

Recently, *Z. galactanivorans* Dsij^T^ has been comprehensively studied and has become a model organism for polysaccharide degradation investigation among marine flavobacteria [30]. The genus *Zobellia* was created by Barbeyron et al. [31], and to date it contains five validly described representatives: *Z. galactanivorans* Dsij^T^, *Z. uliginosa* DSM 2061^T^, *Z. amurskyensis* KMM 3526^T^, *Z. laminariae* KMM 3676^T^, and *Z. russellii* KMM 3677^T^, which were isolated from diverse ecological niches. Although many isolates have also demonstrated an ability to degrade different polysaccharides [32], little is known about the genomic organization of hydrolytic systems within the *Zobellia* genus.

In this study, we performed de novo genome sequencing of two type *Zobellia* strains to produce the first genomics analysis of the genus and provide insights into the role of the CAZyme genomic repertoire in the degradation potential of marine bacteria. Some polysaccharide degradation systems received particular attention due to their biotechnological and medical applications.

## 2. Results and Discussion

### 2.1. Genome Sequencing and Assembly

Among five currently described *Zobellia* species, there are four genomes available to the public on the NCBI database as of October 2019: *Z. galactanivorans* Dsij^T^ (PRJEB8976), *Z. galactanivorans* OII3 1c (PRJNA377409), *Z. uliginosa* DSM 2061^T^ (PRJNA329763), and *Z. amurskyensis* MAR 2009 138 (PRJNA248513, revised from “*Z. uliginosa* MAR 2009 138”). However, only two genomes were obtained from type strains. We obtained two novel draft genomes of the other *Zobellia* species, deposited in the collection of marine microorganisms (KMM WDCM644) of PIBOC FEB RAS.

Type strains *Z. amurskyensis* KMM 3526^Т^ and *Z. laminariae* KMM 3676^Т^ were isolated from seawater (Amur Bay, Vladivostok, Russia) and brown alga *Laminaria japonica* (Troitsa Bay, Zarubino, Russia), respectively, and validly described by Nedashkovskaya et al. [33]. Draft genomes of both flavobacteria were obtained using Roche-454 technology; additionally, the genome of *Z. laminariae* was produced using Ion Torrent technology. De novo genome assembly was performed using trimmed high-quality sequencing reads (>Q20). Assembly statistics are presented in Table 1. The total amount of genomic data on average provided more than 15-fold coverage per genome.

Genome assemblies were validated by remapping sequencing reads back to contigs using the Bowtie2 program (Table 2). The number of reads aligned exactly one time exceeded 95%, and more than once did not exceed 1%, which reflected the high accuracy of assemblies. It is shown that the combination of the two sequencing technologies enables a higher-quality version of the genome assembly to be obtained. Therefore, the draft genomes of *Z. amurskyensis* and *Z. laminariae* were obtained in sufficient quality for the subsequent bioinformatics analysis.

### 2.2. Phylogenetic Analysis

**Figure 1:**
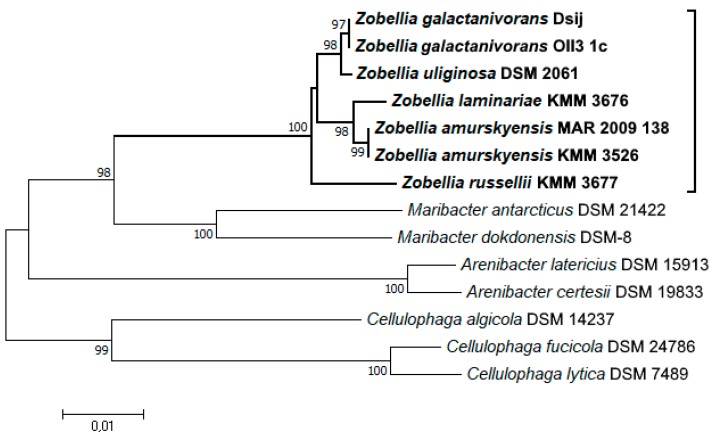
Phylogenetic relationships of *Zobellia* species and representatives of the related genera of the family *Flavobacteriaceae*, based on 16S rRNA gene sequence comparisons. The phylogenetic tree was constructed using the neighbor-joining (NJ) approach [34] with bootstrap support of 1000 replications. The scale bars represent 0.01 substitutions per site.

A phylogenetic tree of the *Zobellia* genus including all type strains and representatives of related genera was inferred based on 16S rRNA partial sequences, which were retrieved from genomic sequences and a nucleotide sequence for *Z. russellii* KMM 3677^T^. According to the neighbor-joining (NJ) tree (Figure 1), all *Zobellia* clustered together and three subclades could be distinguished. One subclade included *Z. uliginosa* and strains of *Z. galactanivorans*, while the other subclade included *Z. laminariae* and strains of *Z. amurskyensis*. This clustering indicates a closer sequence similarity of the strains within subclades. Interestingly, *Z. russellii* branched deeply within the *Zobellia* clade and demonstrated significant evolutionary divergence from all other strains in the genus, supported by high bootstrap values.

In order to clarify in detail the phylogenetic relationships of *Zobellia* species based on obtained and known draft genomes, further phylogenomic measures were performed using the JSpecies Web Server (JSpeciesWS; http://jspecies.ribohost.com/jspeciesws/). JSpeciesWS is a web service for in silico calculation of the extent of identity between genomes. The service measures the average nucleotide identity (ANI) based on BLASTþ (ANIb) and MUMmer (ANIm), as well as correlation indexes of tetranucleotide (Tetra) signatures [35].

The ANI and Tetra values were calculated and are summarized in Table 3. Consistent with the NJ tree, the genomes of *Z. galactanivorans* OII3 1c and *Z. amurskyensis* MAR 2009 138 strains showed ANI values above 97% with their corresponding type strains, which clearly matched the recommended cut-off point for species delineation of ∼96% ANI [36]. Some discrepancies between ANI and Tetra values were observed for *Z. uliginosa*. Although Tetra signatures were in range >0.989, implying that *Z. uliginosa* is closely related to strains of *Z. galactanivorans*, the estimated ANI values of 92%–94% were slightly lower than the species delineating threshold. Therefore, these strains could either belong to the same species from which *Z. uliginosa* recently diverged, or they are two discrete, albeit closely related, species.

### 2.3. Comparative Genomics

Since *Z. galactanivorans* DsiJ^T^ and *Z. galactanivorans* OII3 1c represent the same species, the genome of strain OII3 1c was excluded from the analysis. However, despite the ANI values, the genome of *Z. amurskyensis* MAR 2009 138 was taken into comparative analysis along with a novel draft genome of the type strain KMM 3526^Т^.

Gene prediction and preliminary annotation of *Z. amurskyensis* and *Z. laminariae* genomes were performed with the Rapid Annotation using Subsystems Technology (RAST) server (http://rast.theseed.org/FIG/rast.cgi). In addition to the identification of genes, RAST groups annotated genes into functional subsystems represented by 27 categories of well-characterized metabolic processes and structural complexes [37,38,39]. Based on such data, we could estimate the contribution of diverse metabolic processes to bacterial life strategies. The total number of protein-coding sequences of 4248 and 4334 accounted for KMM 3526^Т^ and KMM 3676^Т^ genomes, among which only 2683 and 2699 genes were functionally annotated, respectively. According to the server, about 1500 genes for both flavobacteria are in subsystems, among which “Carbohydrates” was ranked first in gene content.

Genome characteristics of *Z. amurskyensis* and *Z. laminariae* in comparison to publicly available *Zobellia* genomes are shown in Table 4. Genome sizes ranged slightly within 5.14 Mb to 5.52 Mb. Estimated GC content ranged from 36.77% in *Z. laminariae* to 42.8% in *Z. galactanivorans*. It is worth noting that the comparison was made between draft genomes, for which reason overall metrics strongly depend on genome assembly completeness and annotation methods. Since the obtained genomes were annotated using RAST, other genomes from NCBI were also passed through the RAST server for further comparative analysis.

**Figure 2:**
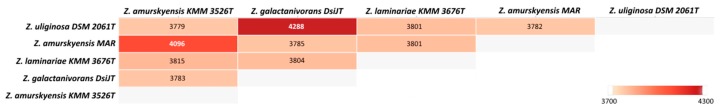
The pairwise heatmap of overlapping cluster numbers across the genomes.

Genome-wide exploration of orthologous genes/clusters across different species is important in comparative genomics to understand molecular evolution, structure of genes and genomes, as well as adaptive capabilities [40]. Orthologs or orthologous genes originate by vertical descent from a single gene in the last common ancestor [41]. Comparison and annotation of orthologous clusters between five *Zobellia* genomes were performed using the web server OrthoVenn2 (https://orthovenn2.bioinfotoolkits.net/home) [42]. Inferred proteins for each genome by RAST annotation were used as input. Consistent with phylogenomic analysis, the pairwise heatmap (Figure 2) demonstrates the phylogenetic proximity of *Z. galactanivorans* to *Z. uliginosa* at the ortholog level.

**Figure 3:**
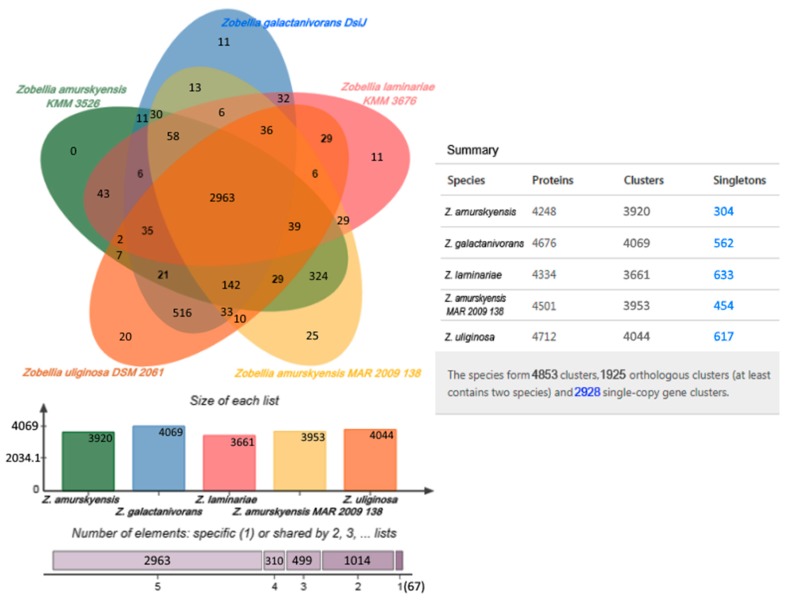
The Venn diagram plotted by OrthoVenn2 shows shared orthologous protein clusters among the genomes of five *Zobellia* strains. The numbers of shared and unique genes, singletons are shown.

The Venn diagram is widely used to visualize similarities and differences between genomes. The distribution of shared orthologous clusters and singletons for each strain is depicted in Figure 3. Singletons are genes for which no orthologs could be found in other species; single-copy gene clusters are clusters that contain single-copy genes in each species [42]. According to cluster analysis, the genomes shared 4853 clusters constituting a supposed pan-genome of the *Zobellia* genus. The core-genome represented in all strains was estimated in 2963 clusters whose functions were mostly assigned to the cellular metabolic process.

From Figure 3, it is apparent that 516 orthologous clusters composed of 1044 genes were represented only in *Z. galactanivorans* and *Z. uliginosa* genomes, while the genomes of *Z. amurskyensis* KMM 3526^Т^ and *Z. amurskyensis* MAR 2009 138 shared 324 clusters of 658 genes. Such clusters are presumably species-specific. Gene ontology (GO) analysis revealed an enrichment of GO:0005983 “starch catabolic process” in both groups. The group of 516 clusters had additionally GO:0016139 “glycoside catabolic process” and the second group had GO:0008484 “sulfuric ester hydrolase activity”.

The dispensable genome of the *Zobellia* genus is composed of singletons or inparalogs, which were unique to each strain. The *Z. laminariae* genome contained the highest number of unique genes, including 633 singletons and 11 clusters of 26 inparalogs. For *Z. uliginosa*, *Z. galactanivorans,* and *Z. amurskyensis* MAR 2009 138 617/562/454 singletons and 48/23/58 inparalogs, respectively, were identified. In the genome of *Z. amurskyensis* KMM 3526^Т^, there were only 304 singletons. These accessory genes possibly affect metabolic differences within *Zobellia* representatives and determine peculiarities of lifestyle in certain ecological niches, such as sediment, seaweeds, or seawater. However, it should be noted that these differences also could be explained by different completeness of the genomes.

### 2.4. Repertoire of CAZymes

We focused on investigation and comparison of the CAZymes genes in the *Zobellia* genomes in order to speculate about their bacterial lifestyles, as well as to identify relevant CAZymes for potential application in medicine and biotechnology.

CAZymes are a class of enzymes that synthesize, modify, or break down saccharides, and their classification comprises the following modules: Glycoside hydrolase families (GHs), polysaccharide lyase families (PLs), carbohydrate esterase families (CEs), glycosyltransferase families (GTs), auxiliary activity families (AAs), and carbohydrate-binding module (CBM) families [13].

A genomic approach was used to explore all CAZymes of a genome (CAZome) more profoundly. Identification of CAZymes across *Zobellia* genomes was carried out using the dbCAN2 meta server (http://cys.bios.niu.edu/dbCAN2). The server allows us to make a more accurate prediction of the CAZome because it integrates three annotation tools: HMMER, DIAMOND, and Hotpep searches [43]. The proportions of CAZymes predicted in the genomes of *Zobellia* are shown in Table 5. Calculations were based on the data obtained by RAST gene prediction and dbCAN2 CAZyme annotation.

**Figure 4:**
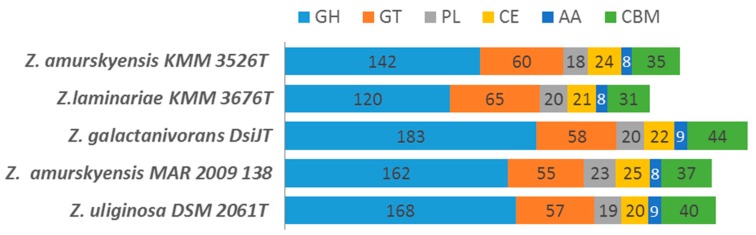
Carbohydrate-active enzymes in *Zobellia* species. GH, glycoside hydrolase; GT, glycosyltransferase; PL, polysaccharide lyase; CE, carbohydrate esterases; AA, auxiliary activities; CBM, carbohydrate-binding module.

As discussed by Barbeyron et al. [30] and Boncan et al. [14], a CAZome is characteristic of species, which gives insights into bacterial behavior, lifestyle, and ecological niche. Therefore, for free-living species the proportion of CAZymes in their genomes typically corresponds to 1%–5% of all predicted coding sequences. In the five *Zobellia* strains studied, the proportion of CAZymes in the genomes ranged from 5.93% in *Z. laminariae* KMM 3676^Т^ to 6.74% in *Z. galactanivorans* DsiJ^T^, indicating the ability to consume various polysaccharides. Other *Zobellia* had slightly lower proportion of CAZymes than *Z. galactanivorans* DsiJ^T^, these values being sufficient to argue that a broad biodegradation potential is conserved at the genus level. Total statistics of CAZymes’ classes predicted across the genomes are in Figure 4.

**Figure 5:**
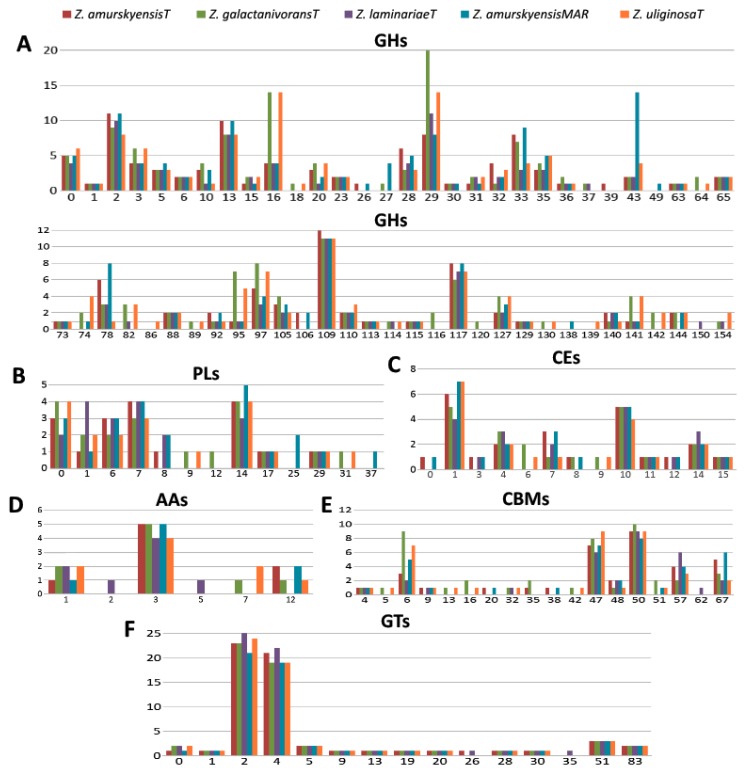
Number of CAZymes in the *Zobellia* species. Number of (**A**) GH families; (**B**) PL families; (**C**) CE families; (**D**) AA families; (**E**) CBM families; (**F**) GT families; GHs, glycoside hydrolases; PLs, polysaccharide lyases; AAs, Auxiliary Activities; CBMs, carbohydrate-binding modules; GTs, glycosyltransferases.

The determination of core and pan CAZomes for the *Zobellia* genus is of particular interest and importance. Obviously, the core CAZomes are composed of genus-specific enzymes, while the enzymes identified in singletons and inparalogs are species-specific. In terms of lifestyle peculiarities, the most interesting are the core multigenic CAZyme families. According to this idea, the core and pan CAZomes of the *Zobellia* genus were determined, and the repertoire of CAZymes is summarized in Figure 5 and Table S1.

GHs are enzymes that catalyze the hydrolytic cleavage of the glycosidic bond between two or more carbohydrates or between a carbohydrate and a non-carbohydrate moiety. These enzymes are involved in the degradation of the majority of biomass, including seaweeds [44]. In the present study, a total of 775 GHs were classified into 62 families in five *Zobellia* genomes. Among the identified core glycoside hydrolases, the most dominant were the GH29, GH109, GH2, GH13, and GH117 families in order of abundance. It is worth noting that seven particular GH13 subfamilies—GH13_11, GH13_19, GH13_3, GH13_31, GH13_38, GH13_7, and GH13_9—were predicted. Based on the CAZy database (http://www.cazy.org/) definitions, enzymes of predicted families might act as broad spectrum α-fucosidases, α-N-acetylgalactosaminidase, β-glycosidases with Koshland double-displacement mechanism, as well as glycosidases acting on substrates with α-glucoside linkages, and α-1,3-L-(3,6-anhydro)-galactosidases.

GTs are principal enzymes that catalyze oligosaccharide, polysaccharide, and glycoconjugate synthesis. They also assist in glycosyl group transfer to specific acceptor molecules and utilize various sugar-1-phosphate derivatives [45]. A total of 16 GT families including 297 GTs were identified for the strains. The GT2 and GT4 families were ranked as key glycosyltransferases for the genus, which are polyspecific enzymes.

PLs are a group of enzymes that cleave uronic acid-containing polysaccharides via a β-elimination mechanism [46]. In the *Zobellia* genomes, a total of 100 PLs were classified in 13 families, among which PL14 lyases, possessing alginate, exo-oligoalginate, and β-1,4-glucuronan lytic activities, were the most abundant.

CEs are a class of esterases that catalyze the de-O or de-N-acylation of substituted saccharides [47]. There are two core multigenic families, namely CE1 and CE10, with wide substrate specificities, which generally help to degrade substrates leading to saccharification [48].

CBMs are non-catalytic proteins with carbohydrate-binding activity, capable of binding carbohydrate ligands and enhancing the catalytic efficiency of other CAZymes [49]. In the present study, a total of 186 CBMs were classified into 18 families, among which three multigenic families (CBM6, CBM47, CBM50) were identified in *Zobellia* genus.

AAs are the last class created in the CAZy classification, comprising enzymes that break glycosidic bonds via an oxidation mechanism [50]. Today, CAZy lists 16 AA families of enzymes playing a significant role in the degradation of biopolymers (CAZy database; http://www.cazy.org/). CAZyme annotation revealed that there are six different AA families in *Zobellia* strains: AA1, AA2, AA3, AA5, AA7, and AA12. The majority of AAs are AA3 with up to five AA3 family members in individual genomes. Moreover, this enzyme group was observed in all studied *Zobellia* strains, while other families were less populated (from zero to two AAs per genome).

### 2.5. Phylogenetic Analysis of Biotechnologically Relevant Cazymes

#### 2.5.1. Polysaccharide-Degrading GH Systems

A closer inspection of the CAZyme genomic repertoires for four *Zobellia* species (Figure 5 and Table S1) revealed representatives of some GH families targeting red and brown algal polysaccharides, namely four (*Z. amurskyensis* and *Z. laminariae*) to 14 (*Z. galactanivorans* and *Z. uliginosa*) GH16 enzymes, including β-agarases, β-porphyranases, laminarinases and κ-carrageenases; one (*Z. uliginosa*) to two (*Z. galactanivorans*) GH64 laminarinases; one (*Z. laminariae*) to three (*Z. galactanivorans* and *Z. uliginosa*) GH82 ι-carrageenases; six to seven (*Z. galactanivorans*, *Z. laminariae*, and *Z. uliginosa*) to eight to nine (strains of *Z. amurskyensis*) GH117 α-1,3-(3,6-anhydro)-L-galactosidases; two to three (*Z. amurskyensis* and *Z. laminariae*) to four (*Z. galactanivorans* and *Z. uliginosa*) GH127 α-1,3-(3,6-anhydro)-D-galactosidases. All five *Zobellia* genomes encode for one GH129 α-1,3-(3,6-anhydro)-D-galactosidase, and only *Z. laminariae* has one enzyme assigned to GH50 β-agarase. No representatives from the other agarolytic enzymes GH86, GH96, GH118, or GH150 were identified.

Previously, *Z. galactanivorans* has been extensively investigated in degradation of various algal polysaccharides through genomic and transcriptomic analysis combined with computer modeling and experimental validation [30,51,52,53,54]. Therefore, the majority of key genes of agar, laminarin, and carrageenan utilization systems of *Z. galactanivorans* can serve as reference sequences for the annotation of hydrolytic enzymes from other *Zobellia* genomes. Our analysis showed that the genomes of *Z. galactanivorans* and *Z. uliginosa* shared the largest reservoir of agarolytic genes among *Zobellia* genomes. Their polysaccharide-degrading systems were represented by GH16 enzymes, including four to five β-porphyranases PorA-E, four to five β-agarases AgaA-D, three to four laminarinases LamA-D, and one κ-carrageenases CgkA. Based on the phylogenetic analysis (Figure S1) of the GH16 catalytic module, the enzymatic systems of other *Zobellia* species were represented by only PorD (Zam_1698, Zmar_1649, and Zlam_2939), PorB (Zam_2877, Zmar_2570, and absent in *Z. laminariae*), AgaC (Zam_3480, Zmar_956, and absent in *Z. laminariae*), AgaB (Zam_3011, Zmar_1702, and Zlam_2991), and LamB (Zlam_4246, absent in *Z. amurskyensis*). The genome of *Z. laminariae* also codes Zlam_2677 as a new putative GH16 subfamily, which occupies an intermediate position on the tree, between the branches CgkA and LamA. The orthologues genes for AgaA and AgaD, as well as for PorA and PorC, which encode secreted enzymes responsible for the initial attack on agars and porphyrans [51,55], were absent in both *Z. amurskiensis* and *Z. laminariae* genomes. Therefore, PorD, PorB, AgaC, and AgaB, as well as LamB are the genus-specific GH16 enzymes, potentially possessing broader substrate specificities. It has been recently shown that *Z. galactanivorans* AgaC, defined as a new GH16 subfamily, can hydrolyze not only agarose, but also complex agars [56]. Interestingly, the *Z. uliginosa* genome encodes two strongly different AgaC sequences, classical AgaC Zuli_2505 and AgaC-like Zuli_8, which can be of a great biotechnological interest because it is a new β-agarase. Therefore, the *Zobellia* β-agarases, which play a key role in agar depolymerization with the release of a range of neoagarooligosaccharides, are likely to be considered for use in industrial and biotechnological applications.

The *Zobellia* genomes contained the multigenic GH117 family coding exolytic 3,6 anhydro-α-L-galactosidases, which cleave neoagarooligosaccharides and produce L-AHG, and therefore perform a key role in terminal steps of polysaccharide saccharification. Previously, the products of some GH117 genes of *Z. galactanivorans* (Zga_4663 (ZgAhgA), Zga_3615, and Zga_3597) were biochemically and structurally characterized [57]. The multigenic GH117 families consisted of six (*Z. galactanivorans*), seven (*Z. uliginosa* and *Z. laminariae*) or eight (*Z. amurskiensis*) genes. Our phylogenetic analysis (Figure S2) is in agreement with the previously obtained GH117 tree [58], with the exception of the additional clades formed by GH117 of *Z. amurskiensis* strains: Clade 8 (ZamT_1387 and ZamMar_2539) and Clade 9 (ZamT_1385 and ZamMar_2537). We consider these additional clades of GH117 enzymes to reflect new enzymatic specificities.

Analysis of the genomic regions around GH16 and GH117 genes revealed a number of potential GH2 β-galactosidase genes. Recently, a novel agarolytic GH2 β-galactosidase has been found in the marine bacterium *Vibrio* sp. EJY3 [59]. Therefore, we suggested that these GH2s might be exo-β-1,4-galactosidases, removing galactose at the non-reducing end of agarooligosaccharides. Previously, a similar genomic sequence containing several GH2s, GH16s, and GH117s was identified as a putative agarolytic cluster in the human intestinal bacterium *Bacteroides uniformis* Bu NP1 [60]. It was suggested that the products of GH16s were cyclically degraded into monosaccharides by the coordinated work of GH117B and GH2C, respectively.

Since there is a demand for highly specific agarolytic enzymes, the investigation of multigenic families encoding enzymes with slightly different activities and specificities may be the best solution for production of valuable oligosaccharides and rare monomers with different bioactivities or applications.

#### 2.5.2. Auxiliary Activity Family 3 Enzymes

According to the CAZyme annotation (Figure 5 and Table S1), AA3 is characterized by a multiplicity of members (up to five candidate proteins) for the *Zobellia* genus. AA3 belongs to the glucose-methanol-choline (GMC) oxidoreductase family first outlined by Cavener [61]. It was reported that GMC oxidoreductases were flavoproteins containing FAD-binding domain with the strictly conserved Rossmann fold or β-α-β dinucleotide-binding motif GXGXXG [62]. Our results demonstrate that proteins in the studied *Zobellia* strains predicted as AA3 enzymes also have such motif, suggesting that they may act as GMC oxidoreductases (Figure S3).

The GMC oxidoreductases are a very large and functionally diverse enzyme superfamily divided into four subfamilies, which include cellobiose dehydrogenases, glucose oxidoreductases, aryl-alcohol oxidases, alcohol oxidases, and pyranose oxidoreductases [63]. In 2012, Kim et al. [64] identified GMC oxidoreductase from a *Rhizobium* sp. strain GIN611 with glycoside deglycosylation activity different from that of common glycosidases (GHs). Later [65] they characterized its homologs in *Stenotrophomonas maltophilia*, *Sphingobacterium multivorum*, and *Agrobacterium tumefaciens* strains, catalyzed the deglycosylation via the same mechanism, and suggested these enzymes as a new GMC oxidoreductase subfamily—FAD-dependent glycoside oxidoreductase (FAD-GO). Interestingly, the authors showed broad glycone and aglycon specificities for these enzymes that makes them very attractive in their industrial applications.

We performed a phylogenetic analysis by comparing the amino acid sequences of *Zobellia* AA3 members with characterized FAD-GOs. According to the phylogenetic tree (Figure S4), the *Zobellia* enzymes formed two clades, one of which (Clade A) was orthologous to the FAD-GO proteins. Sequence comparison of *Zobellia* Clade A enzymes and glycoside oxidoreductases revealed relatively high identity between them (53%–58%) (Table S2). Moreover, His493 residue considered as a catalytic FAD-GO amino acid for an initial oxidation step was observed within protein sequences for all studied *Zobellia* (Figure S3). This gives us the opportunity to suggest that *Zobellia* AA3 enzymes could have the same glycoside oxidase activity, identification of which may be the subject for further research. Due to the broad substrate specificity, putative *Zobellia* FAD-GOs are of particular interest and can be considered as promising biocatalysts for glycoside deglycosylation in food and pharmaceutical industries [65].

## 3. Materials and Methods

### 3.1. Genome Sequencing and Assembly

Genomic DNA was isolated from stationary phase cultures of *Z. amurskyensis* KMM 3526^Т^ and *Z. laminariae* KMM 3676^Т^ using a NucleoSpin kit (Macherey-Nagel, Düren, Germany) following manufacturer’s instructions. The quantity and quality of isolated DNA were analyzed using NanoPhotometer Pearl (IMPLEN, Munich, Germany). The shotgun DNA libraries were constructed according to the methodological recommendations described in the GS Junior Titanium Rapid Library Preparation Method Manual, GS Junior Titanium emPCR Amplification Method Manual—Lib-L, GS Junior Titanium Sequencing Method Manual, NEBNext^R^ dsDNA Fragmentase^R^, NEXTflex™ DNA-Sequencing Kit for Ion Platforms, KAPA Library Quantification Kit Ion Torrent™ Platforms, Ion 520™ & Ion 530™ Kit-Chef. Libraries of both flavobacteria were sequenced on the 454 GS Junior (454 Life Sciences, Branford, CT, USA); additional library of the KMM 3676^Т^ was sequenced at Far Eastern Federal University, School of Biomedicine, on the Ion Torrent IonS5 XL platform (Thermo Fisher Scientific, Waltham, MA, USA). All sequencing reads were preprocessed with FastQC (https://www.bioinformatics.babraham.ac.uk/projects/fastqc/) and Prinseq (http://edwards.sdsu.edu/cgi-bin/prinseq/prinseq.cgi) to remove the adaptor sequences and low-quality data. A de novo assembly of filtered reads was performed using Newbler version 3.0 (454 Life Sciences, Branford, CT, USA and SPAdes version 3.11.1 [66]; validation of an assembly was done by remapping filtered reads to the contigs by using Bowtie2 (Galaxy version 2.3.4.3+galaxy0) [67]; metrics were calculated with the help of QUAST (Galaxy version 5.0.2+galaxy0) [68].

### 3.2. Genome Annotation

Gene prediction and automated genome annotation were carried out using Rapid Annotation using Subsystem Technology (RAST) v. 2.0 with default parameters [37,38,39] followed by manual curation of the some annotations by comparing translated sequences with the NCBI non-redundant database, InterPro (https://www.ebi.ac.uk/interpro/), and Pfam (https://pfam.xfam.org/) databases. For more accurate annotation of carbohydrate-active enzymes, their classification into existing CAZy families and identification of a CAZome repertoire of *Zobellia* genus were performed using the dbCAN2 meta server (http://cys.bios.niu.edu/dbCAN2) [43].

### 3.3. Phylogenetic, PhylogenomicAnalyses, and Comparative Genomics

Phylogenetic analysis of 16S rRNA gene sequences, also members of GHs and AAs from *Zobellia* species, was performed using the NJ [34] method with bootstrap supporting of 1000 replicates in MEGA v.6.06 [69]. Phylogenomic measures were calculated with the JSpecies Web Server [35] to determine ANI values and Tetra signatures. Genome-wide analysis of orthologous clusters and gene ontology analysis among all predicted protein-coding genes was performed using OrthoVenn2 (https://orthovenn2.bioinfotoolkits.net/home) [42].

### 3.4. Deposition of the Nucleotide Sequence Accession Number

The whole-gGenome shotgun sequences of *Z. amurskyensis* KMM 3526^Т^ and *Z. laminariae* KMM 3676^Т^ have been deposited at DDBJ/ENA/GenBank under the accessions RCNR00000000 and RCNS00000000, respectively. The versions described in this paper are RCNR01000000 and RCNS01000000.

## 4. Conclusions

Today, some of the most eco-friendly methods for obtaining algal polysaccharides and their derivatives are enzyme-based techniques. Therefore, the search for marine bacteria specialized in the degradation of various polysaccharides is of particular interest. The marine flavobacterium *Z. galactanivorans* Dsij^T^ is a model organism for polysaccharide degradation investigation among marine flavobacteria. However, little has been known about the genomic basics of hydrolytic potential of the *Zobellia* genus. To determine the CAZyme content at the species and genus taxonomic levels, we performed genome sequencing of two type *Zobellia* strains and comparative genomic analysis. We identified a relatively high proportion of CAZymes in the genomes of five *Zobellia* strains. Our comparative study strongly suggests a specialization of the *Zobellia* genus in the algal polysaccharide degradation. These microorganisms can be used as both strain-degraders and valuable sources of novel enzymes for potential application in biotechnology, food, and medical industries.

## Figures and Tables

**Table 1:** Genome assembly statistics of two *Zobellia* strains.

Criteria	*Z. amurskyensis* KMM 3526^Т^	*Z. laminariae* KMM 3676^Т^
Total number of aligned bases	79,609,284	386,897,826
Total number of contigs	157	35
Number of contigs > 1 kb	100	17
Number of contigs > 0.5 kb	110	24
Lengths of the longest contig, bp	221,511	1,629,023
N50, bp	94,524	1,429,896
N75, bp	46,058	1,415,858
L50	17	2
L75	37	3
Coverage	16	75

**Table 2:** Assembly validation metrics.

Criteria	*Z. amurskyensis* KMM 3526^Т^	*Z. laminariae* KMM 3676^Т^
Filtered reads	251,270	2,482,522
Aligned 0 times (%)	10,951 (4.36)	44,386 (1.79)
Aligned exactly 1 time (%)	239,721 (95.40)	2,417,097 (97.36)
Aligned >1 times (%)	598 (0.24)	21,039 (0.85)
Overall alignment rate (%)	95.64	98.21

**Table 3:** Results of average nucleotide identity (ANI; %) and tetranucleotide (Tetra) calculations using JSpecies Web Server (JSpecies WS).

	ANIb/ANIm, %	1	2	3	4	5	6
Tetra							
**1.***Z. amurskyensis* KMM 3526^Т^			77.59/ 83.14	83.80/ 86.97	97.40/ 98.27	77.56/ 83.16	77.51/ 83.18
**2.***Z. galactanivorans* DsiJ^T^		0.78805		77.00/ 82.80	77.58/ 83.15	98.69/ 99.37	92.91/ 94.02
**3.***Z. laminariae* KMM 3676^Т^		0.98333	0.72544		83.92/ 86.83	76.88/ 82.83	76.84/ 82.54
**4.***Z. amurskyensis* MAR 2009 138		0.99923	0.7949	0.98223		77.60/ 83.21	77.45/ 83.10
**5.***Z.galactanivorans* OII3 1c		0.792	0.99968	0.72942	0.799		92.94/ 94.06
**6.** *Z. uliginosa*		0.78333	0.99905	0.71978	0.7902	0.99887	

**Table 4:** Comparison of the genome characteristics of *Zobellia* strains.

Features	*Z. amurskyensis* KMM 3526^Т^	*Z. laminariae* KMM 3676^Т^	*Z. galactanivorans* DsiJ^T^	*Z. amurskyensis* MAR 2009 138	*Z. uliginosa* DSM 2061^T^
Genome size, Mb	5.142451	5.159845	5.52171	5.358000	5.303163
GC Contents, %	38.02	36.77	42.80	38.10	42.60
CDS (by RAST)	4248	4334	4676	4501	4712
CDS (by NCBI)	-	-	4515	4339	4356

**Table 5:** Proportions of predicted carbohydrate-active enzymes (CAZymes) in the genomes of *Zobellia* strains.

Strain	No. of genes	No. of CAZymes	% CAZymes
*Z. amurskyensis* KMM 3526^Т^	4248	276	6.49
*Z. laminariae* KMM 3676^Т^	4334	257	5.93
*Z. galactanivorans* DsiJ^T^	4676	315	6.74
*Z. amurskyensis* MAR 2009 138	4501	299	6.64
*Z. uliginosa* DSM 2061	4712	296	6.28

## Supplementary Materials

The following are available online at https://www.mdpi.com/1660-3397/17/12/661/s1, Table S1: Repertoire of CAZymes of the *Zobellia* genus; Figure S1: Phylogenetic tree of *Zobellia* GH16 proteins; Figure S2: Phylogenetic tree of *Zobellia* GH117 proteins; Figure S3: Multiple amino acid sequence alignment of *S. maltophilia*, *A. tumefaciens*, *S. multivorum*, and *Zobellia* strain AA3 enzymes (GMC-oxidoreductases); Figure S4: Phylogenetic tree of *Zobellia* AA3 proteins and *S. maltophilia*, *A. tumefaciens*, *S. multivorum* FAD-GOs. Table S2: Comparison of amino acid identities (%) of *Zobellia* AA3 enzymes with *S. maltophilia*, *A. tumefaciens*, *S. multivorum* characterized FAD-GOs.
