# Supplementary material for: Comparative Genomics and CAZyme Genome Repertoires of Marine Zobellia amurskyensis KMM 3526T and Zobellia laminariae KMM 3676T
Source: Mar Drugs. 2019 Nov 24;17(12):661. doi: 10.3390/md17120661 (PMC6950322; doi:10.3390/md17120661)
Supplement: Supplementary file 1 [file marinedrugs-17-00661-s001.pdf]

Supplementary materials

"Comparative Genomics and CAZyme Genome Repertoires of Marine *Zobellia amurskyensis* KMM 3526^{T} and *Zobellia laminariae* KMM 3676^{T}"

Nadezhda Chernysheva[1], Evgeniya Bystritskaya[1], Anna Stenkova[2], Ilya Golovkin[2], Olga Nedashkovskaya[1] and Marina Isaeva[1*]

[1] G.B. Elyakov Pacific Institute of Bioorganic Chemistry, Far Eastern Branch, Russian Academy of Sciences, 159, Pr. 100 let Vladivostoku, Vladivostok 690022, Russia; chernysheva.nadezhda@gmail.com (N.C.); belyjane@gmail.com (E.B.); oined2012@gmail.com (O.N.).

[2] Far Eastern Federal University, 8 Sukhanova St., Vladivostok 690090, Russia; stenkova@gmail.com (A.S.); golovkin.io.1996@gmail.com (I.G.).

[*] Correspondence: issaeva@gmail.com (M.I.); Tel.: +7-914-702-0915

Submitted to Marine Drugs.

Table S1. Repertoire of CAZymes of the *Zobellia* genus.

GH	0	1	2	3	5	5_13	5_2	5_4	5_42	6	10	13	13_11	13_19	13_3	13_31	13_38	13_7	13_9	15	16
*Z. amurskyensis*T	5	1	11	4	1	0	0	1	1	2	3	2	1	1	1	1	1	1	2	1	4
*Z. galactanivorans*T	5	1	9	6	0	0	1	1	1	2	4	1	1	1	1	1	1	1	1	2	14
*Z. laminariae*T	4	1	10	4	0	0	1	1	1	2	1	1	1	1	1	1	1	0	2	2	4
Z. amurskyensis MAR	5	1	11	4	1	1	0	1	1	2	3	2	1	1	1	1	1	1	2	1	4
*Z. uliginosa*T	6	1	8	6	0	0	1	1	1	2	1	1	1	1	1	1	1	1	1	2	14

GH	18	20	23	26	27	28	29	30_4	31	32	33	35	36	37	39	43	43_1	43_12	43_17	43_18	43_26
*Z. amurskyensis*T	0	3	2	1	0	6	8	1	1	4	8	3	1	0	1	0	2	0	0	0	0
*Z. galactanivorans*T	1	4	2	0	1	3	20	1	2	1	7	4	2	1	0	0	2	0	0	0	0
*Z. laminariae*T	0	1	2	0	0	4	11	1	2	2	3	3	1	1	0	0	2	0	0	0	0
Z. amurskyensis MAR	0	2	2	1	4	5	8	1	1	2	9	5	1	0	0	6	2	1	1	1	1
*Z. uliginosa*T	1	4	2	0	0	3	14	0	2	3	4	5	1	0	0	1	2	1	0	0	0

GH	43_31	43_9	49	63	64	65	73	74	78	82	86	88	89	92	95	97	105	106	109	110	113	114
*Z. amurskyensis*T	0	0	0	1	0	2	1	0	6	0	0	2	0	2	1	5	3	2	12	2	1	0
*Z. galactanivorans*T	0	0	0	1	2	2	1	2	3	3	0	2	1	1	7	8	4	0	11	2	1	1
*Z. laminariae*T	0	0	0	1	0	2	1	0	3	1	0	2	0	1	1	3	2	0	11	2	1	1
Z. amurskyensis MAR	1	1	1	1	0	2	1	1	8	0	0	2	0	2	1	4	3	2	11	2	1	0
*Z. uliginosa*T	0	0	0	1	1	2	1	4	1	3	1	2	1	1	5	7	2	0	11	3	1	1

GH	115	116	117	120	127	129	130	138	139	140	141	142	144	150	154	GT	0	1	2	4	5	9
*Z. amurskyensis*T	1	0	8	0	2	1	0	0	0	2	1	0	2	0	0		1	1	23	21	2	1
*Z. galactanivorans*T	1	2	6	1	4	1	1	0	0	1	4	2	2	0	1		2	1	23	19	2	1
*Z. laminariae*T	1	0	7	0	2	1	0	0	0	2	1	0	0	1	1		2	1	25	22	2	1
Z. amurskyensis MAR	1	0	8	0	3	1	0	1	0	2	1	0	2	0	0		1	1	21	19	2	1
*Z. uliginosa*T	1	0	7	0	4	1	1	0	1	1	4	2	2	0	2		2	1	24	19	2	1

GT	13	19	20	26	27	28	30	35	51	83	PL	0	1_2	6	7	7_3	7_5	8_2	9_4	12_3	14	14_3
*Z. amurskyensis*T	1	1	1	1	0	1	1	0	3	2		3	1	3	2	0	2	1	0	0	4	0
*Z. galactanivorans*T	1	1	1	0	0	1	1	0	3	2		4	2	2	1	1	1	0	1	1	3	1
*Z. laminariae*T	1	1	1	1	0	1	1	1	3	2		2	4	3	2	0	2	2	0	0	3	0
Z. amurskyensis MAR	1	1	1	0	0	1	1	0	3	2		3	1	3	2	0	2	2	0	0	5	0
*Z. uliginosa*T	1	1	1	0	0	1	1	0	3	2		4	2	2	1	1	1	0	1	0	3	1

PL	17_2	25	29	31	37	CE	0	1	3	4	6	7	8	9	10	11	12	14	15	AA	1	2
*Z. amurskyensis*T	1	0	1	0	0		1	6	1	2	0	3	1	0	5	1	1	2	1		1	0
*Z. galactanivorans*T	1	0	1	1	0		0	5	0	3	2	1	1	1	5	1	0	2	1		2	0
*Z. laminariae*T	1	0	1	0	0		0	4	1	3	0	2	0	0	5	1	1	3	1		2	1
Z. amurskyensis MAR	1	2	1	0	1		1	7	1	2	0	3	1	0	5	1	1	2	1		1	0
*Z. uliginosa*T	1	0	1	1	0		0	7	0	2	1	1	0	1	4	1	0	2	1		2	0

AA	3	5_2	7	12	CBM 4	5	6	9	13	16	20	32	35	38	42	47	48	50	51	57	62	67
*Z. amurskyensis*T	5	0	0	2	1	0	3	1	0	0	1	0	1	1	0	7	2	9	0	4	0	5
*Z. galactanivorans*T	5	0	1	1	1	1	9	0	1	2	0	1	2	0	1	8	1	10	2	2	0	3
*Z. laminariae*T	4	1	0	0	1	0	2	1	0	0	0	1	0	0	0	6	2	9	0	6	1	2
Z. amurskyensis MAR	5	0	0	2	1	0	5	1	0	0	1	0	0	1	0	7	2	8	1	4	0	6
*Z. uliginosa*T	4	0	2	1	1	1	7	1	1	1	0	1	0	0	1	9	1	9	1	3	0	2

The entire table corresponds to the pan CAZome and columns representing core CAZymes are colored in light gray. The most abundant enzyme families, which include many members both genus- and species-specific, are in gray.

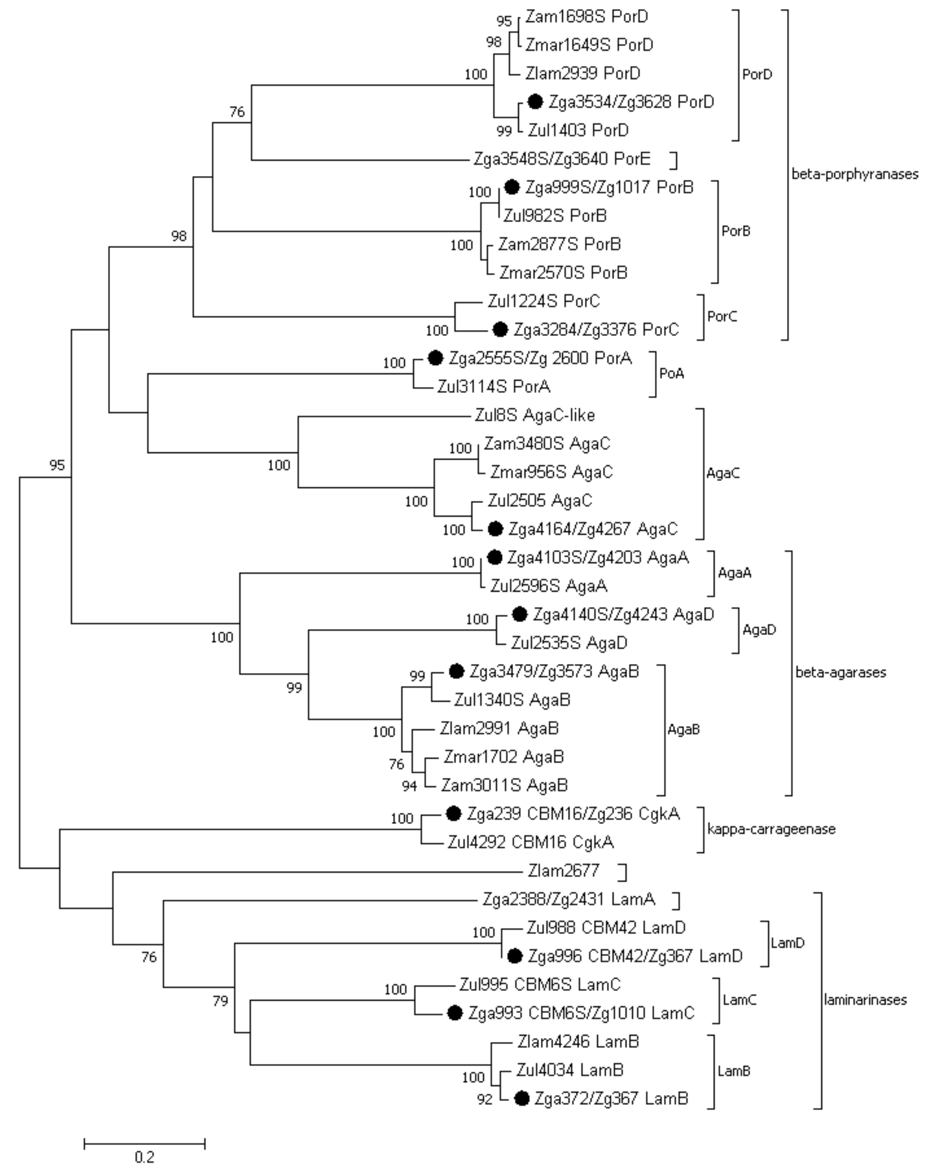

Figure S1. Phylogenetic tree of *Zobellia* GH16 proteins. The phylogenetic tree was constructed by the neighbor-joining approach [34] with bootstrap support of 1000 replications. The scale bars represent 0.2 substitutions per site.

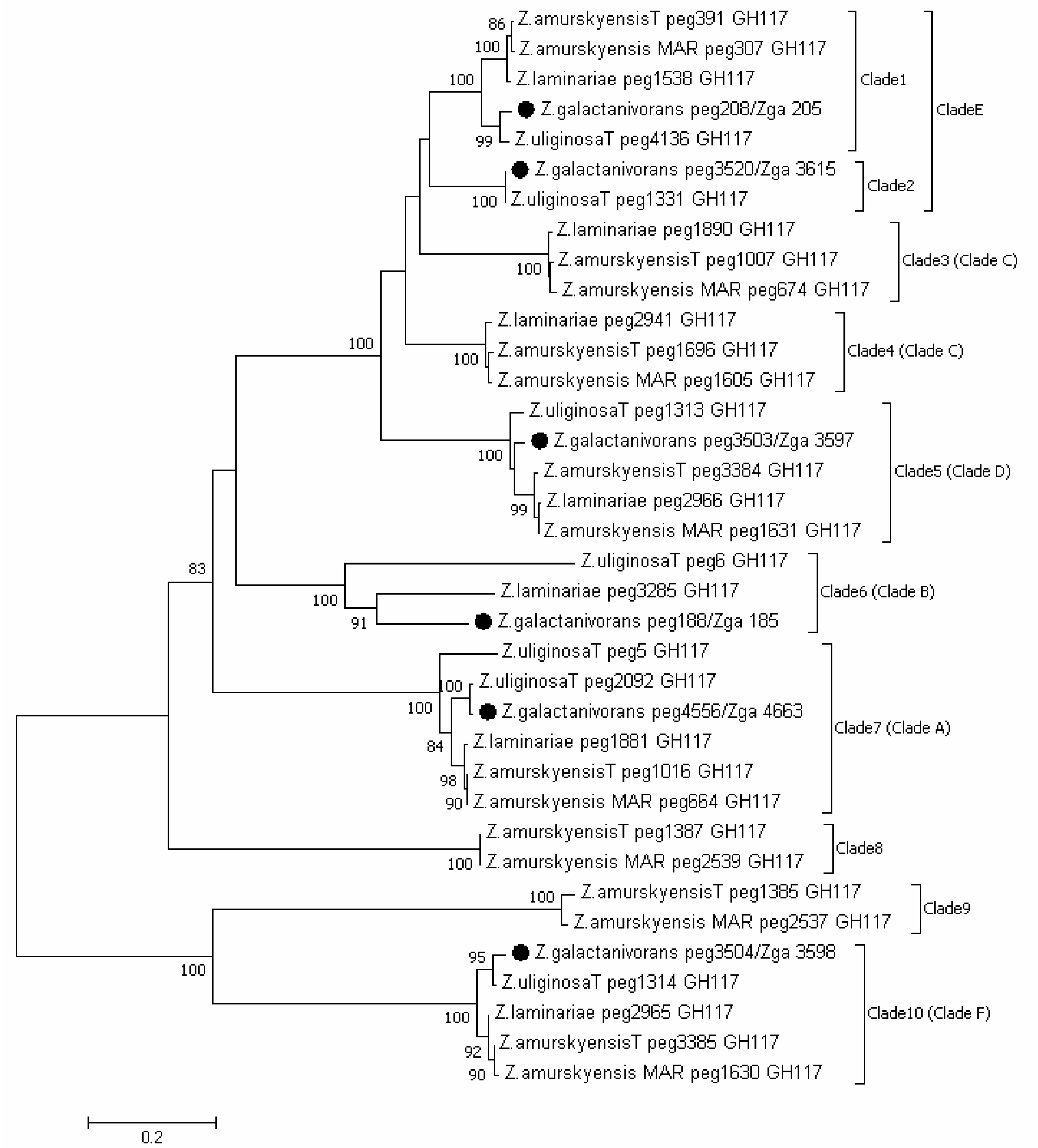

Figure S2. Phylogenetic tree of *Zobellia* GH117 proteins. The phylogenetic tree was constructed by the neighbor-joining approach [34] with bootstrap support of 1000 replications. The scale bars represent 0.2 substitutions per site.

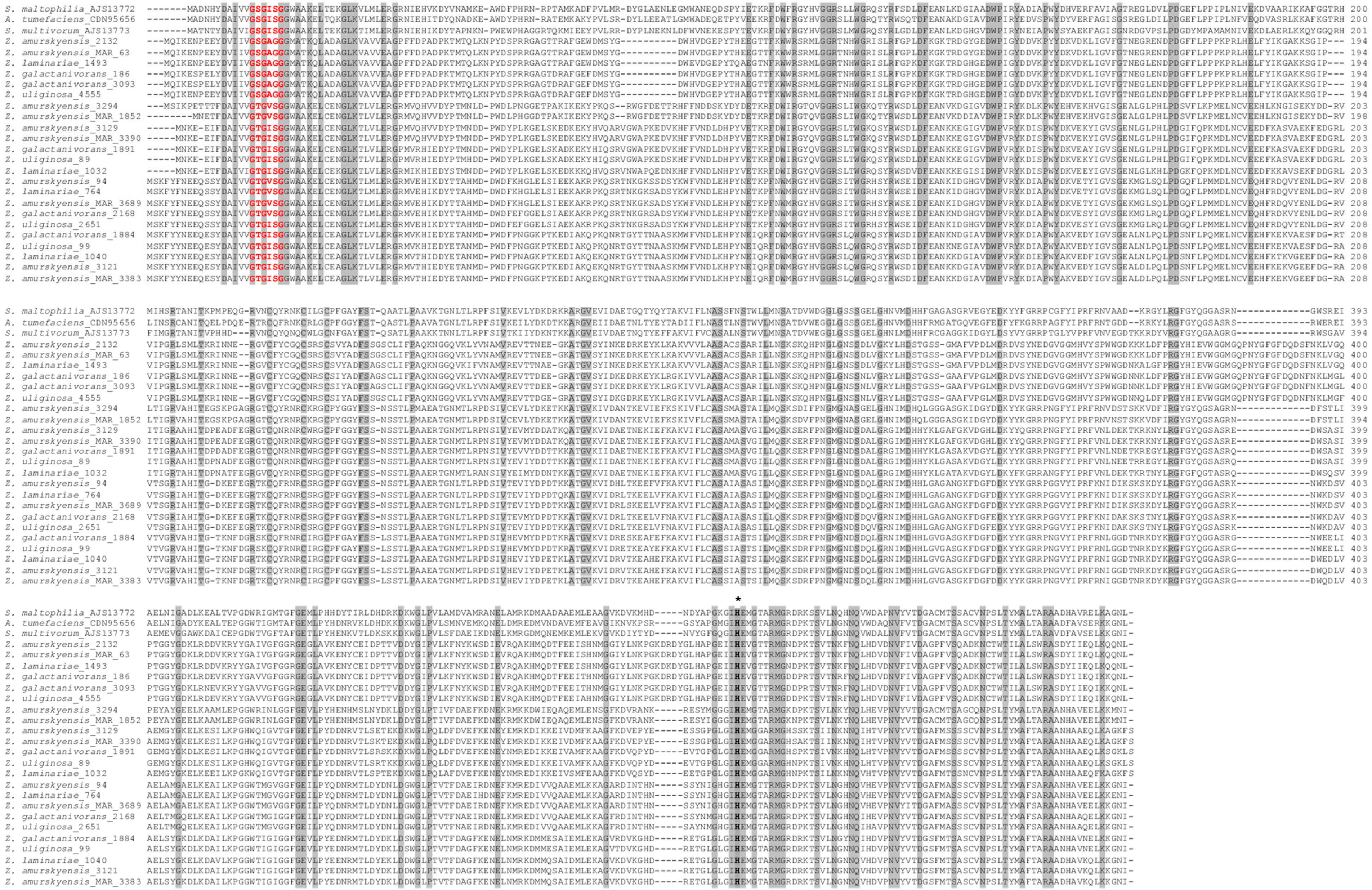

Figure S3. Multiple amino acid sequence alignment of *S. maltophilia*, *A. tumefaciens*, *S. multivorum*, and *Zobellia* strain AA3 enzymes (GMC-oxidoreductases). Identical residues are indicated by light grey color. Conserved β-α-β dinucleotide-binding motif of FAD-binding domain is shown by red color letters. Catalytic residue (His) for oxidation of glycone mojety is highlited in dark grey and marked with an asterisk.

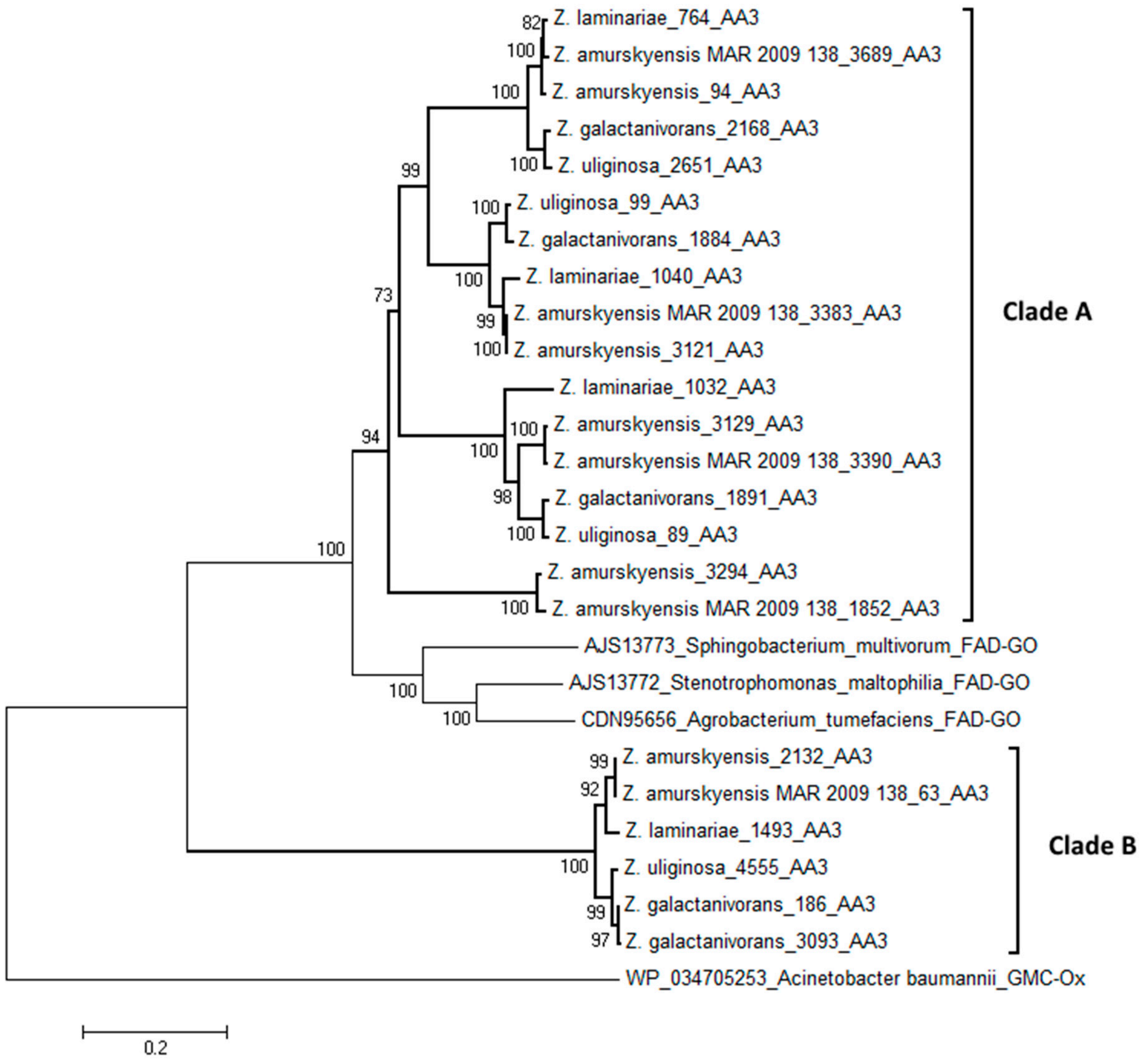

Figure S4. Phylogenetic tree of *Zobellia* AA3 proteins and *S. maltophilia*, *A. tumefaciens*, *S. multivorum* FAD-GOs. *Acinetobacter baumanii* GMC-oxidoreductase was used as outgroup. The phylogenetic tree was constructed by the neighbor-joining approach [34] with bootstrap support of 1000 replications. The scale bars represent 0.2 substitutions per site.

Table S2. Comparison of amino acid identities (%) of *Zobellia* AA3 enzymes with *S. maltophilia, A. tumefaciens*, *S. multivorum* characterized FAD-GOs

	S. maltophilia AJS13772	*A. tumefaciens* CDN95656	*S. multivorum* AJS13773	*Z. amurskyensis*_2132	*Z. amurskyensis*_MAR 2009 138 63	*Z. laminariae*_1493	*Z. galactanivorans*_186	*Z. galactanivorans*_3093	*Z. uliginosa*_4555	*Z. amurskyensis*_3294	*Z. amurskyensis*_MAR 2009 138 1852	*Z. amurskyensis*_3129	*Z. amurskyensis*_MAR 2009 138 3390	*Z. galactanivorans*_1891	*Z. uliginosa*_89	*Z. laminariae*_1032	*Z. amurskyensis*_94	*Z. laminariae*_764	*Z. amurskyensis*_MAR 2009 138 3689	*Z. galactanivorans*_2168	*Z. uliginosa*_2651	*Z. galactanivorans*_1884	*Z. uliginosa*_99	*Z. laminariae*_1040	*Z. amurskyensis*_3121	*Z. amurskyensis*_MAR 2009 138 3383
S. maltophilia AJS13772		77	66	30	30	30	30	30	30	54	54	54	54	54	54	54	56	55	55	55	55	58	58	57	58	58
A. tumefaciens CDN95656			67	30	30	29	30	30	30	54	54	54	54	55	55	55	55	55	55	55	55	57	57	56	57	57
S. multivorum AJS13773				31	31	31	31	30	30	54	54	53	53	53	53	54	54	54	54	53	54	56	56	56	56	56
